# An investigation of the smoking behaviours of parents before, during and after the birth of their children in Taiwan

**DOI:** 10.1186/1471-2458-8-67

**Published:** 2008-02-20

**Authors:** Shu-Fang Shih, Likwang Chen, Chi Pang Wen, Wei-Chih Yang, Yaw-Tang Shih

**Affiliations:** 1 Institute of Health Policy and Management, College of Public Health, National Taiwan University, 6F, No.17, Hsu-Chow Rd., Taipei 100, Taiwan; 2 Center for Health Policy Research and Development, National Health Research Institutes, No.35 Keyan Road, Zhunan Town, Miaoli County 350, Taiwan; 3 Institute of Public Health & Department of Social Medicine, School of Medicine, National Yang-Ming University, Taipei City 112, Taiwan; 4 Institute of Healthcare Administration, College of Health Science, Asia University, No. 500, Lioufong Road, Wufong Shiang, Taichung 41354, Taiwan

## Abstract

**Background:**

Although many studies have investigated the negative effects of parental smoking on children and Taiwan has started campaigns to promote smoke-free homes, little is known about the smoking behaviours of Taiwanese parents during the childbearing period. To help fill the gap, this study investigated Taiwanese parents' smoking behaviours before, during and after the birth of their children, particularly focusing on smoking cessation during pregnancy and relapse after childbirth.

**Methods:**

We used data from the Survey of Health Status of Women and Children, conducted by Taiwan's National Health Research Institutes in 2000. After excluding survey respondents with missing information about their smoking behaviours, our sample consisted of 3,109 women who were married at the time of interview and had at least one childbearing experience between March 1, 1995 and February 28, 1999. Data on parental smoking behaviour in the six months before pregnancy, during pregnancy, and in the first year after childbirth were extracted from the survey and analysed by descriptive statistics as well as logistic regression.

**Results:**

Four percent of the mothers and sixty percent of the fathers smoked before the conception of their first child. The educational attainment and occupation of the parents were associated with their smoking status before the first pregnancy in the family. Over 80% of smoking mothers did not quit during pregnancy, and almost all of the smoking fathers continued tobacco use while their partners were pregnant. Over two thirds of the women who stopped smoking during their pregnancies relapsed soon after childbirth. Very few smoking men stopped tobacco use while their partners were pregnant, and over a half of those who quit started to smoke again soon after their children were born.

**Conclusion:**

Among Taiwanese women who had childbearing experiences in the late 1990s, few smoked. Of those who smoked, few quit during pregnancy. Most of those who quit relapsed in the first year after childbirth. The smoking prevalence was high among the husbands of these Taiwanese women, and almost all of these smoking fathers continued tobacco use while their partners were pregnant. It is important to advocate the benefits of a smoke-free home to Taiwanese parents-to-be and parents with young children, especially the fathers. The government should take advantage of its free prenatal care and well-child care services to do this. In addition to educational campaigns through the media, the government can request physicians to promote smoke-free homes when they deliver prenatal care and well-child care. This could help reduce young children's health risks from their mothers' smoking during pregnancy and second-hand smoke at home.

## Background

Increasing health costs and loss of productivity due to tobacco related diseases are well known results of tobacco use; expenditure on tobacco may also use up money that should be used on essential expenditure for the family [1]. Moreover, evidences have shown that parental smoking can have negative impacts on their children's physical health, particularly in their children's embryo stage and infancy. For instance, maternal tobacco use or exposure to second-hand smoke can cause low birth-weight and premature death, and inhaling environmental tobacco smoke (ETS) can cause sudden infant death syndrome (SIDS) [2,3]. In spite of the harmful effects of parental smoking, previous studies have indicated that a substantial proportion of pregnant women still smoke [4,5]. For instance, 13% of pregnant women smoked in Sweden in 2000 [6], and 12% of pregnant women smoked in the USA in 2000 [7]. Most of such studies in the literature explored situations in Western countries. Most research exploring parental tobacco use also focuses on maternal smoking behaviours. In general, the literature has shown that most women do not quit smoking during their pregnancies, and the postpartum relapse rate is high among those who do [8-11].

Of the few studies investigating paternal smoking behaviours, a German study using data from a national health survey conducted in 1984–1986 found that paternal smoking behaviours were much less affected by the birth of their children, compared to maternal smoking behaviour [12]. A qualitative study in Australia showed that fathers were largely unaware of health risks to their unborn children from their smoking, and also lacked motivation to quit smoking during their wives' pregnancies [13]. One British study surveying men with very young children found that only 20% of these fathers had tried to quit tobacco use since the birth of their children, and only 4% of them were successful in that attempt [14]. Some studies in this field explore the relationship between mothers' smoking cessation and their partners' smoking status, and showed that pregnant women and mothers with young children were more likely to be smokers and less likely to quit smoking, if they had smoking partners [15,16]. This indicates that parental and maternal smoking behaviours are correlated.

This study explores the patterns of smoking cessation and subsequent relapse of Taiwanese parents with young children. Taiwan is a country with a high smoking prevalence rate for men and a relatively low rate for women. In 1996, 1999, 2001, and 2004, the smoking prevalence rates of male adults (aged 18 or over) in Taiwan were 51.9%, 47.3%, 46.5%, and 43.8%, respectively [17,18]. The smoking prevalence of Taiwanese women (aged 18 or over) has been around 3 to 5% for the last twenty years [17,18]. Over the past decade, Taiwan has had many anti-smoking campaigns. However, it did not start putting much effort into promoting smoke-free homes until a couple of years ago. So far, there has also been little information regarding Taiwanese parents smoking behaviours before, during, and after the birth of their children. Furthermore, little is known with respect to factors associated with these smoking patterns, and the relationship between paternal and maternal smoking behaviours in these periods.

To help fill the gap, this study investigated these issues, putting special focus on parental smoking cessation during pregnancy and relapse after childbirth. This is the first study to examine these issues in Taiwan. Specifically, we estimated Taiwanese parents' smoking prevalence rates at different times over the childbearing period, including the six months before each pregnancy, during each pregnancy, and the infancy of each of their first three children. We also investigated the parents' tendencies to quit smoking during pregnancy and to relapse in the first year after childbirth, and explored whether the parents' socio-economic status and their partners' smoking status were associated with their smoking status and smoking cessation behaviour. Findings from this study can highlight the need for governmental policy in this regard not only for Taiwan but also for countries in a similar development stage.

## Methods

### Data

This study used data from the Survey of Health Status of Women and Children, a national face-to-face interview survey conducted by Taiwan's National Health Research Institutes in 2000. It is the only survey that can provide detailed information about Taiwanese parents' smoking behaviours over the childbearing period. Although it was conducted seven years ago and can provide data only for 2000 and earlier years, findings from analysing the data are still valuable and constructive for policy making, as the female smoking rate in Taiwan has been quite stable over the past twenty years and the male smoking prevalence is still high while having a declining trend.

This retrospective survey adopted a "probability proportional to size" stratified sampling method and collected data for about one sixth of all townships in Taiwan. The response rate was 76%. The respondents were the main caregivers of a group of Taiwanese children born between March 1, 1995 and February 28, 1999. Over 98% of the responding caregivers were the children's mothers. In total, the survey collected data for 7,817 children in 3,934 families. The children formed a national representative sample of children born between March 1 of 1995 and February 28 of 1999 as well as their siblings.

While the main study purpose of this survey was to investigate young Taiwanese children's utilization of well-child care, the survey database also contains information about parental smoking behaviour over their childbearing period, which we used for this present study. In case that the respondent was the mother and she was married at the time of the interview, she was asked to report her and her partner's smoking status over the childbearing period. After excluding survey respondents with missing information about their smoking behaviours, our sample consisted of 3,109 women who were married at the time of interview with each bearing at least one child between March 1, 1995 and February 28, 1999.

For each of these women's children who were alive at the time of the interview, this survey collected information on the mother's and the father's smoking status in the six months before the mother conceived the child, during this pregnancy, and in the year right after the birth of this child. These children's birthdays were not confined to the period between March 1, 1995 and February 28, 1999. Among a mother's children alive at the time of the interview, each child's birth order was reported, and this birth order was used as the proxy for the actual birth order.

### Statistical analyses

The first two parts of our analyses were descriptive. First, we analysed smoking status for men and for women with young children. We estimated the smoking prevalence rates in 2000 for these parents. For each single childbirth experience, we also estimated the smoking prevalence rates of both the parents in the six months before the pregnancy, during the pregnancy, and in the first year after the childbirth.

Second, we investigated parental tendencies to quit smoking during pregnancy and to restart smoking in the first year after childbirth. In such analysis, only parents smoking in the six months before conceiving a child were included in the sample. We did such an analysis for both mothers and fathers, and for each of the first two childbirth experiences. As mentioned previously, a child's birth order out of the mother's children still living at the time of the interview was used as the proxy for this child's actual birth order. Because Taiwan has a low infant mortality rate (smaller than 7 per thousand in the 1990s) and a low mortality rate for children (smaller than 0.6 per thousand for children aged 1–4, and smaller than 0.3 per thousand for children aged 5–14 in the 1990s) [19], this proxy was very close to the actual birth order.

The final part of our analyses was based on multivariate analysis. We examined the relationships of socio-economic status with a parent's smoking status in the six months before the first child was conceived, and this parent's tendency to quit smoking for at least some time after the first child was conceived. We also explored how a parent's smoking status in the six months before the first child was conceived and this parent's tendency to quit smoking for at least some time after the first child was conceived were related to the partner's smoking status. Logistic regression was used for this part of analysis.

For smoking status in the six months before the first pregnancy, the outcome variable was a binary variable assigned the value of 1 if the parent smoked during the six months, and 0 otherwise. For smoking cessation after the first child was conceived, the outcome variable was a binary variable assigned the value of 1 if the parent quit smoking for at least some time, and 0 if not. The major explanatory factors were a parent's educational attainment and occupation, monthly family income, and the smoking status of this parent's partner in the six months before the first pregnancy experience of the family. The residential region and urbanization level of the area of residence were also included as control variables.

We used the odds ratio to show the magnitudes of associations of the aforementioned outcome variables with socio-economic status and the partner's smoking status. The Huber/White/sandwich estimator was employed to obtain robust variance estimates. This method is a commonly used estimator of standard errors, and it is robust without assuming that the standard errors are independent from the explanatory variables and are identically distributed.

## Results

### Sample characteristics

At the time of interview, 17% of these parents (518 in 3,109) had one child, 54% (1,691) had two children, and 29% had three or more children (900). The proportions of the mothers with junior high school education or less, of those having senior high school education, and of those having at least college education were 19%, 54%, and 27%, respectively. For the fathers, the proportions corresponding to the three educational levels were 20%, 45%, and 35%, respectively. The proportions of the mothers having high-position (professionals, entrepreneurs, managers or administrative employees), middle-position (small shop owners, clerks, or skilled workers), and low-position (non-skilled workers or unemployed persons) occupations were 13%, 29%, and 58%, respectively. For the fathers, the proportions of high-, middle-, and low-position occupations were 29%, 44%, and 27%, respectively. For 2000, 11% of the families earned less than 30,000 Taiwanese dollars per month. The proportions corresponding to 30,000–49,999, 50,000–69,999, and 70,000 or more were 32%, 28%, 29%, respectively.

### Smoking status for men and for women with young children over their childbearing period

The overall smoking prevalence rate for women in general in 2000 was 4.2%; 6.2% for those with one child, 4.0% for those with two, and 3.6% for those with three or more. Women with one child had a higher smoking prevalence rate than their counterparts with more children, but not statistically significant (p > 0.05). The overall smoking prevalence rate for the men in 2000 was 58.1%. The rates for men with one child, those with two, and those with three or more were 56.2%. 56.4% and 62.2%, respectively. Men with three or more children had a higher smoking prevalence rate than their counterparts with fewer children (p < 0.01).

Table 1 shows these men's and women's smoking status at different times over their childbearing period. These results show that these parents' smoking status at the time of the interview was very similar to what it was during the six months prior to the first pregnancy experience of the family. About 60% of the fathers smoked during and after their wives' pregnancy. About 4% of them smoked during and after pregnancy.

**Table 1:** Parental smoking prevalence rates at different times over the childbearing period

	Smoking prevalence rate			
	
	Mothers		Fathers	
		
Period	Smokers	%	Smokers	%
*First child*	N = 3,109		N = 3,109	
In the six months right before this pregnancy	129	4.15	1862	59.89
During this pregnancy	103	3.31	1844	59.31
In the child's infancy	122	3.92	1845	59.34
*Second child*	N = 2,591		N = 2,591	
In the six months right before this pregnancy	90	3.47	1548	59.75
During this pregnancy	78	3.01	1539	59.40
In the child's infancy	94	3.63	1546	59.67
*Third child or later*	N = 1,072		N = 1,071	
In the six months right before this pregnancy	44	4.10	692	64.61
During this pregnancy	37	3.45	687	64.15
In the child's infancy	44	4.10	687	64.15

### The tendencies to quit smoking during pregnancy and to restart smoking in the first year after childbirth

Results in Table 2 indicate that few of the smoking mothers quit tobacco use during pregnancy. Table 3 shows that almost no smoking fathers (about 1%) quit smoking while their partners were pregnant. These results also reveal that most parents who refrained from smoking while their children in the embryo stage relapsed during their children's infancy. For the mothers, the relapse rate was 69.2% after the birth of the first child, and 91.7% after the second child. For the fathers, the relapse rates after the first and the second childbirths were 52.6% and 80.0%, respectively.

**Table 2:** Smoking cessation rate during pregnancy

	The first pregnancy				The second pregnancy			
		
	Mothers		Fathers		Mothers		Fathers	
				
Smoking cessation	n	%	n	%	n	%	n	%
Quitting	26	20.16	19	1.02	12	13.33	10	0.68
Not quitting	103	79.84	1843	98.98	78	86.67	1538	99.32

Total	129		1862		90		1548	

**Table 3:** Smoking relapse rate in the first year after childbirth

	The first pregnancy				The second pregnancy			
		
	Mothers		Fathers		Mothers		Fathers	
				
Smoking relapse	n	%	n	%	n	%	n	%
Relapsing	18	69.23	10	52.63	11	91.67	8	80.00
Not relapsing	8	30.77	9	47.37	1	8.33	2	20.00

Total	26		19		12		10	

### The relationships of socio-economic status with a parent's smoking status in the six months before the first child was conceived, and this parent's tendency to quit smoking for at least some time after the first child was conceived

Results in Table 4 show that educational attainment and occupation were associated with a person's smoking status in the six months before his or her first child was conceived. Due to exclusion of parents with missing values for some explanatory variables, the samples for the multivariate analyses are a little smaller than their corresponding samples for the descriptive analyses. A high-position occupation and high education were associated with a lower probability of being a smoker before the childbearing period. In particular, women with high school education were much more likely to be a smoker than their counterparts with at least some college education (odds ratio: 6.29). In addition, women having a low-position occupation were significantly more likely to be a smoker than their counterparts with a high-position occupation (odds ratio: 5.18). These results also provide some evidence that the family income level was related to the smoking status before the first pregnancy experience of the family. Generally speaking, women with the highest family income level (70,000 Taiwanese dollars or more per month) were more likely to be smokers, but men with the highest family income level were less likely to be smokers.

**Table 4:** Odds ratios for being a smoker in the six months before the first child was conceived, with 95% confidence intervals, for different socio-economic statuses and the partner's smoking status

	Mothers		Fathers	
		
	Odds ratio	95% CI	Odds ratio	95% CI
*Educational attainment (reference group: college or above)*				
Elementary school or below	3.36**	(1.52 7.40)	2.27**	(1.87 2.76)
High school	6.29**	(2.64 14.96)	3.01**	(2.30 3.94)
*Occupation (reference group: high position)*				
Middle position	1.92	(0.54 6.81)	1.46**	(1.19 1.79)
Low position	5.18*	(1.49 18.03)	1.43**	(1.11 1.85)
*Average monthly family income (reference group: NT$70,000 or above)*				
<= 29,999	0.61	(0.34 1.08)	1.22	(0.99 1.52)
30,000 – 49,999	0.42**	(0.23 0.76)	1.27*	(1.01 1.60)
50,000 – 69,999	0.54	(0.26 1.11)	1.30	(0.93 1.82)
*Partner's smoking status before the conception of the first child (reference group: non-smoker)*				
Smoker	6.40**	(3.31 12.37)	5.94 **	(3.02 11.67)

Number of observations	2,837		2,772	
Model Significance: Wald χ^2^(13)	131.80**		257.95**	

** p < 0.01; * p < 0.05.

Note: In the models, the residential region and the urbanization level of residential district were also controlled for.

In general, no evidence was found to support that socio-economic status was associated with a parent's tendency to quit smoking for at least some time after the first child was conceived. The only exception was that fathers with the highest family income level were more likely to quit tobacco use after his first child was conceived than their less well-off counterparts (results not shown, but available upon request from the authors).

### The relationships of the smoking status of a parent's partner with the parent's smoking status in the six months before the first child was conceived and this parent's tendency to quit smoking for at least some time after the first child was conceived

In the six months before a woman's first child was conceived, she was more much likely to be a smoker if her partner smoked. The odds ratio for such women relative to her non-smoking counterparts was 6.40 (Table 4). Similarly, before a man's partner conceived the first child, he was much more likely to smoke cigarettes if his partner smoked, with an odds ratio relative to his non-smoking counterparts being 5.94 (Table 4). However, no evidence was found to support that a parent's tendency to quit tobacco use for at least some time after the family's first child was conceived was associated with his or her partner's smoking status before the first pregnancy experience of the family.

## Discussion

Our findings show that around 60% of the fathers smoked during and after their partners' pregnancy, and around 4% of the mothers smoked during and after pregnancy. The prevalence rates were similar to those for all men aged 25 to 44 and for all women in this age range in 2001 – 56% and 5% [17,20]. In Taiwan, adults of this age range have substantially higher smoking prevalence than those of other age ranges [17,20]. Unfortunately, people of this age range are also more likely to be parents with young children. Moreover, our results suggest that adults in this age range have similar smoking prevalence, regardless of whether they have young children or not. This also suggests that the existence of young children at home does not influence Taiwanese adults' decision to smoke or not. Thus, it is imperative for the government to pay more attention to smoking cessation programs for people with young children, given that second-hand smoke can substantially increase health risks for young children, and parents with young children in Taiwan are not less likely to be smokers than other adults.

Our findings of low smoking cessation rates in Taiwanese adults in the childbearing period are consistent with the literature. Previous studies have highlighted several possible motives for smoking cessation, such as family pressure, social pressure, concern for the health of own offspring, and health effects on others [21,22]. Regarding concern for the health of one's own offspring, some research has suggested that pregnancy should be a critical time to promote smoking cessation, both to women and their partners [1,22]. However, such opportunity is far from well exploited, as the literature and our findings indicate unsatisfactory smoking cessation rates for pregnant women in several European countries, the United States, and Taiwan. It certainly calls for more research to design effective smoking cessation programs for pregnant women and their partners.

Our findings with respect to the high smoking relapse rates of Taiwanese parents with young children are also consistent with the literature. This phenomenon deserves attention, and suggests that a smoking cessation program should not neglect follow-up examinations for participants who successfully quit tobacco use during the program. Therefore, Taiwan's government should endeavour to prevent relapse in people who quit during these smoking cessation programs. A Taiwanese program for eliminating tobacco use at home a couple of years ago could be an example. In 2004, Taiwan's Bureau of Health Promotion put much effort into promoting "smoke-free home," and used this topic to join a worldwide program "Quit & Win." According to the evaluation report for this program, the cessation rate for participants one year after joining the program was around 21–22%, which was higher than those for Canada, Japan and United States [23]. However, there has been no further follow-up evaluation for this program. We think that it is critical to have such follow-up evaluation in order to provide the information needed for the design of more effective programs for helping people permanently stop the habit of smoking cigarettes in the future.

The low smoking cessation rate of Taiwanese parents-to-be and the high relapse rate among parents with infants suggest that Taiwanese parents may have little knowledge about the harmful health effects to their children of second-hand smoke and the benefits of their quitting tobacco use for their children. This highlights the necessity of educational campaigns in this regard. Taiwan's government should take advantage of its free prenatal care and well-child care services to educate these people. In addition to educational campaigns through the media, the government should encourage physicians to promote smoke-free homes when they deliver prenatal care and well-child care to help reduce young children's health risks from their mothers' smoking during pregnancy and second-hand smoke at home.

For example, physicians can check a pregnant woman's smoking behaviour and her risk of exposure to ETS. Information on the health consequences of a mother's smoking during pregnancy on her children in the embryo stage, such as low birth-weight and premature death, should be clearly shared with the mother. Knowledge on the harmful effects of a pregnant woman's exposure to ETS on her children should also be taught during prenatal care. In addition to information and knowledge dissemination, advocates aiming to change smoking parent's attitudes should be also emphasized [24].

Well-child care visits are also a good setting for dissemination of information on the harmful effects of ETS and educational campaigns targeting smoking parent's attitudes. Knowledge on various health risks to young children of inhaling ETS, such as SIDS, should be conveyed to parents or childcare givers who take young children to receive well-child care. To strengthen the effects of information dissemination and educational campaigns, it should be helpful to further provide helpline services that can guide a smoking parent on how to successfully quit tobacco use or instruct a non-smoking parent with a smoking partner how to help the partner to stop smoking.

Educational activities for helping smoking parents to quit tobacco use may be more effective if they include both the father and the mother. Previous research has argued that social support could be substantially helpful for reducing tobacco use during pregnancy [25]. Moreover, our results show that a parent's smoking status before the first pregnancy experience of the family was positively correlated to his or her partner's smoking status, and smoking parents-to-be and parents with young children rarely quit tobacco use. Pregnant women should be encouraged to bring their smoking partners to join educational activities aimed at helping smokers quit. As the smoking prevalence rate is high among Taiwanese fathers-to-be and fathers with young children, including pregnant women's smoking partners in such educational activities is particularly important. For smoking women, teaching them to quit smoking and asking them to ask their partners also to quit may be even more important, since such a way may help in the creation of a supportive environment for the couple to quit simultaneously. Designing educational campaigns that can incorporate the influences of social support and pressure, especially from within the family, should be imperative for the government as it seeks to promote smoke-free homes.

The final issue to discuss pertains to the relationships of parental socio-economic status with their smoking status and decision to quit smoking. Because smoking parents-to-be and parents with young children in Taiwan rarely quit tobacco use, it is certainly important to promote smoking cessation to all smoking parents. On the other hand, more effort may be needed for helping parents with lower socio-economic status. Our findings indicate that adults with less education and lower-position occupations were more likely to smoke before the childbearing period, and fathers with high income were more likely to quit smoking after the first pregnancy experience in the family. These findings suggest that educational campaigns advocating smoke-free home should pay more attention to parents with lower socioeconomic status.

This study has two special merits. First, it used data on women's smoking status at different times in their childbearing period. There have been few studies that have examined women's tobacco use in different pregnancies. Such research can offer information on how women's smoking behaviour may differ at different times, and can thus provide more insight into anti-smoking policy formulation. For instance, one research investigating women's smoking status between successive pregnancies reveals that it is necessary to persistently promote anti-smoking attitudes to pregnant women, regardless of whether they previously smoked or not [26]. Findings from our study also indicate such a necessity. The second merit of this study is that it investigated both women's and their partners' smoking behaviours over the childbearing period. By also examining the smoking behaviours of fathers-to-be and fathers with young children, this study demonstrates the importance of promoting smoking cessation among these men to protect young children from the harms of ETS.

There are two major limitations for our study. The first limitation pertains to recall bias, a problem inherent in most retrospective studies using surveys. Nonetheless, we believe that such recall bias in our study should not be serious for two reasons. First, most mothers tended to remember things happening near their pregnancies well, and data we used were from questions about whether a parent was a smoker in a childbearing period near a pregnancy experience in the family, which was not so hard to recollect, compared to how much tobacco they consumed. Recall bias in our case should tend to result in underestimates of smoking prevalence and overestimates of smoking cessation, given that people tend to give socially-desirable answers in such surveys [27,28], but our findings still indicate high smoking prevalence rates and low smoking cessation rates for parents with young children in Taiwan.

The second research limitation is that our data could only show whether a parent smoked or not, but were not able to indicate whether and how much the parent smoked at home. This made us unable to explore the negative health effects of Taiwanese parents on their children in more depth. Nevertheless, our findings are still enlightening for policy making in this area, since our ultimate goal should be to eliminate ETS, given that there is no safe amount of second-hand smoke [2,3].

## Conclusion

Few women who gave birth in Taiwan during the late 1990s smoked. Among those who did, few quit during pregnancy. Most of those who quit during pregnancy relapsed in the first year after childbirth. The smoking prevalence was high among the husbands of these Taiwanese women, and almost all of these smoking fathers continued to smoke while their partners were pregnant. Advocating the benefits of smoke-free home to Taiwanese parents-to-be and parents with young children is certainly imperative, especially to fathers. In addition to educational campaigns through the media, the government can ask physicians to promote smoke-free homes when they deliver prenatal care and well-child care, services offered free of charge in Taiwan, to help reduce young children's health risks from their mothers' smoking during pregnancy and second-hand smoke at home.

## Competing interests

The author(s) declare that they have no competing interests.

## Authors' contributions

SFS participated in the design of the study, led the statistical analysis, interpreted the findings, and also participated in writing the manuscript. LC designed the study, oversaw the statistical analysis, interpreted the findings, and took the major responsibility of drafting the manuscript. CPW participated in interpreting the findings and writing the manuscript. WCY participated in designing the study and analysing the data. YTS participated in the design of the study and helped draft the manuscript. All authors read and approved the final manuscript.

## Pre-publication history

The pre-publication history for this paper can be accessed here:


